# The Story of the Insane from Year to Year

**Published:** 1897-07-31

**Authors:** 


### THE STORY OF THE INSANE FROM YEAR TO YEAR.

Tenth Series.

WORCESTER COUNTY ASYLUM.

During 1896 the numbers were almost stationary, being 1,038 on January 1st and 1,043 on December 31st. There were 184 first admissions, 31 readmissions, 74 recoveries, 10 discharged relieved, and 94 deaths. The recoveries stand at 36·2 per cent. of the admissions, and the deaths at 9 per cent. of the average number resident. Thirty-eight of the deaths were caused by cerebral and spinal diseases, 8 by consumption, 1 by enteritis, and 1 by enteric fever. Various moral causes account for the insanity in 38 of the year's admissions, intemperance in drink for 28, and hereditary influence in 21. The questions put by the Commissioners in Lunacy relative to the real or supposed increase of insanity seem to have stirred up a great many medical superintendents to investigate the subject. Dr. Cooke thinks, with the majority, that there is no real increase, and that accumulations from a diminished death-rate and other causes account for the apparent increase. That much of the increase is to be accounted for in this way is certain; but we doubt whether the whole can be so accounted for. We are, however, entirely in accord with Dr. Cooke in thinking that we have very nearly reached the upward limit in the curableness of insanity, and we are confirmed in this view when we look at the hopeless nature of the majority of cases admitted to our county asylums. The Worcester Asylum is to be once more enlarged, and when the additions are finished it will accommodate 1,200 patients, a number which we believe to be at least 200 too high. There were five cases of enteric fever during the year. Two of these were attacked in January, one in June, and two in August; and Dr. Cooke tells us that nothing pointed to a common cause as to the origin of these cases. We are pleased to congratulate Nurse Hannah Turner on having received her pension, and when it is stated that she served over thirty years there can be no doubt that she deserved it. The average weekly cost of maintenance is 8s. per head.

### THE CLONMEL DISTRICT ASYLUM.

The present accommodation is for 600 patients, but on January 1st there were 676 in residence, and by the end of the year the number had risen to 697. There were 83 first admissions, 18 readmissions, 47 recoveries, and 25 deaths. The recoveries stand at 46 per cent. on the admissions, and the deaths at 6 per cent. on the average number resident. Only 6 of the deaths were caused by cerebral diseases, 6 by consumption, and 2 by intestinal diseases. Domestic trouble, mental anxiety, and such things caused the insanity in 7 of the year's admissions, intemperance in drink in 4, and hereditary influence in 50. To relieve the overcrowding it has been suggested either to build additions or to convert part of an adjoining workhouse to asylum purposes, but neither of these schemes will be carried out until it is seen whether the Irish Poor Law Reform Bill will become law. Dr. Harvey, the assistant medical officer, has given a course of lectures on nursing, and 10 of the attendants have obtained the certificate of the Medico-Psychological Association. The cost of maintenance per head per annum was £23 8s. 1d.

### WONFORD HOUSE, EXETER.

The asylum or hospital contained 123 patients at the beginning of the year, and 121 at the end of it. There were 22 admissions, 7 recoveries, 8 discharged relieved, and 6 deaths. The recoveries stand at 33·3 per cent., and the deaths at 4·9 per cent., both calculated in the way usual in asylums. The recovery rate during the last 13 years has been 46·4, but, as Dr. Deas remarked, when small numbers are under consideration percentages are apt to vary from no cause other than the numbers. The hospital does a considerable amount of charitable work. At the present time one-half of the patients are received at sums lower than the average cost per head, and among these are four who are maintained free and ten who pay under twenty shillings a week. There is in connection with the hospital a seaside residence at Dawlish, and, altogether, it may be said that Dr. Deas does everything he can to promote recovery in the curable and to make asylum residence as pleasant as possible for the incurable.

### BETHLEM HOSPITAL.

During the year the numbers fell from 217 to 211. There were 163 first admissions, 40 readmissions, 103 recoveries, 14 discharged relieved, and 18 deaths. The recoveries stand at 50·2 per cent. on the admissions, and the deaths at 7·9 per cent. on the average number resident, but no comparison can be drawn between Bethlem Hospital and a county asylum because the class of patients differs considerably. Ten of the deaths were from cerebral diseases, 1 from consumption, and 1 from suicide. No blame was attributed to any of the staff in connection with this death. Of the year's admissions 96 are stated to owe their disease to various moral causes alone or in combination with other causes; intemperance in drink in 18 cases; and hereditary influence in 95. We congratulate Nurse Sophia Nevill on having obtained her pension after nineteen years' service. Dr. Smith says that the proposed Lunacy Law Amendment Bill may affect the regulations of hospitals for the insane, and he hopes that nothing more will be added to the already crushing routine entailed by the existing law. We hope so too; but then the last Act was so bad that we should not be surprised at anything.

